# Different solubilizing ability of cyclodextrin derivatives for cholesterol in Niemann–Pick disease type C treatment

**DOI:** 10.1002/ctm2.1350

**Published:** 2023-08-24

**Authors:** Yusei Yamada, Madoka Fukaura‐Nishizawa, Asami Nishiyama, Akira Ishii, Tatsuya Kawata, Aina Shirakawa, Mayuko Tanaka, Yuki Kondo, Toru Takeo, Naomi Nakagata, Toru Miwa, Hiroki Takeda, Yorihisa Orita, Keiichi Motoyama, Taishi Higashi, Hidetoshi Arima, Takahiro Seki, Yuki Kurauchi, Hiroshi Katsuki, Katsumi Higaki, Kentaro Minami, Naoki Yoshikawa, Ryuji Ikeda, Muneaki Matsuo, Tetsumi Irie, Yoichi Ishitsuka

**Affiliations:** ^1^ Department of Clinical Chemistry and Informatics, Graduate School of Pharmaceutical Sciences Kumamoto University Kumamoto Japan; ^2^ Department of Pharmacy University of Miyazaki Hospital Miyazaki Japan; ^3^ Division of Reproductive Engineering, Center for Animal Resources and Development (CARD) Kumamoto University Kumamoto Japan; ^4^ Division of Reproductive Biotechnology and Innovation, Center for Animal Resources and Development (CARD) Kumamoto University Kumamoto Japan; ^5^ Department of Otolaryngology, Graduate School of Medicine Osaka Metropolitan University Osaka Japan; ^6^ Department of Otolaryngology‐Head and Neck Surgery Graduate School of Medicine Kumamoto University Kumamoto Japan; ^7^ Department of Physical Pharmaceutics, Graduate School of Pharmaceutical Sciences Kumamoto University Kumamoto Japan; ^8^ Priority Organization for Innovation and Excellence Kumamoto University Kumamoto Japan; ^9^ Laboratory of Evidence‐Based Pharmacotherapy Daiichi University of Pharmacy Fukuoka Japan; ^10^ Department of Pharmacology, Faculty of Pharmaceutical Sciences Himeji Dokkyo University Hyogo Japan; ^11^ Department of Chemico‐Pharmacological Sciences, Graduate School of Pharmaceutical Sciences Kumamoto University Kumamoto Japan; ^12^ Research Initiative Center, Organization for Research Initiative and Promotion Tottori University Yonago Japan; ^13^ Department of Pediatrics, Faculty of Medicine Saga University Saga Japan; ^14^ Department of Pharmaceutical Packaging Technology, Faculty of Life Sciences Kumamoto University Kumamoto Japan

**Keywords:** cholesterol, cyclodextrin, Niemann–pick disease type C, stoichiometry

## Abstract

**Background:**

Niemann–Pick disease type C (NPC) is a fatal neurodegenerative disorder caused by abnormal intracellular cholesterol trafficking. Cyclodextrins (CDs), the most promising therapeutic candidates for NPC, but with concerns about ototoxicity, are cyclic oligosaccharides with dual functions of unesterified cholesterol (UC) shuttle and sink that catalytically enhance the bidirectional flux and net efflux of UC, respectively, between the cell membrane and the extracellular acceptors. However, the properties of CDs that regulate these functions and how they could be used to improve treatments for NPC are unclear.

**Methods:**

We estimated CD–UC complexation for nine CD derivatives derived from native α‐, β‐, and γ‐CD with different cavity sizes, using solubility and molecular docking analyses. The stoichiometry and complexation ability of the resulting complexes were investigated in relation to the therapeutic effectiveness and toxicity of each CD derivative in NPC experimental models.

**Findings:**

We found that shuttle and sink activities of CDs are dependent on cavity size‐dependent stoichiometry and substituent‐associated stability of CD–UC complexation. The ability of CD derivatives to form 1:1 and 2:1 complexes with UC were correlated with their ability to normalize intracellular cholesterol trafficking serving as shuttle and with their cytotoxicity associated with cellular UC efflux acting as sink, respectively, in NPC model cells. Notably, the ability of CD derivatives to form an inclusion complex with UC was responsible for not only efficacy but ototoxicity, while a representative derivative without this ability negligibly affected auditory function, underscoring its preventability.

**Conclusions:**

Our findings highlight the importance of strategies for optimizing the molecular structure of CDs to overcome this functional dilemma in the treatment of NPC.

## Graphical abstract

1. Cyclodextrins (CDs), the therapeutic candidates for Niemann–Pick disease type C (NPC), function dually as a shuttle and sink that catalytically enhance the cellular cholesterol flux.

2. The shuttle/sink activities of CDs are dependent on cavity size‐dependent stoichiometry and substituent‐associated stability of CD–cholesterol complexation.

3. The shuttle/sink abilities of CDs impact their therapeutic and toxicological properties in NPC treatment.

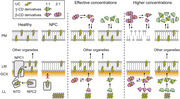

## 1 BACKGROUND

Niemann–Pick disease type C (NPC) is an autosomal recessive lysosomal storage disorder caused by mutations in the *NPC1* (∼95% of affected individuals) or *NPC2* genes.^1^ Following endocytosis, cholesterol derived from low‐density lipoprotein is transferred from lysosomes to other organelles, such as endoplasmic reticulum, through the concerted action of transmembrane NPC1 and luminal NPC2 proteins.^2^ Inherited dysfunction in these proteins disturbs intracellular cholesterol homeostasis, leading to the excessive accumulation of unesterified cholesterol (UC) in lysosomes and the depletion of esterified cholesterol (EC) in other subcellular compartments. Both preclinical models and patients with NPC exhibit progressive neurodegeneration and systemic manifestations, ultimately resulting in premature death.^3^

Cyclodextrins (CDs), cyclic oligosaccharides consisting of six, seven and eight D‐glucopyranose units, called α‐, β‐ and γ‐CDs, respectively, are considered the most promising therapeutic candidates for NPC.^4^, ^5^ Their unique cyclic structure, featuring a hydrophilic outer surface and a hydrophobic inner cavity, provides the ability to form water‐soluble inclusion complexes with guest molecules of an appropriate size and low polarity, such as UC.^6^, ^7^ We and other groups previously suggested that CDs serve dual functions of UC “shuttle” and “sink”, whereby they enhance the bidirectional flux of UC, without changing the equilibrium UC distribution between the plasma membrane and vesicles at lower concentrations while facilitating UC efflux from the plasma membrane and serving themselves as extracellular UC reservoirs at higher concentrations.^8^, ^9^ A β‐CD derivatives with different degrees of substitution (DS) of 2‐hydroxypropyl groups, 2‐hydroxypropyl‐β‐CD (HP‐β‐CD), was discovered serendipitously as a potential therapeutic UC shuttle for NPC.^10^ Although preclinical and clinical studies have demonstrated that peripheral or central administration of HP‐β‐CD attenuates NPC‐related manifestations, unresolved issues, including intolerable adverse effects (e.g. lung injury and hearing loss) and unfavourable physicochemical properties, remain a bottleneck in drug development.^11^, ^12^, ^13^, ^14^, ^15^, ^16^, ^17^

We previously identified that 2‐hydroxypropyl‐γ‐CD (HP‐γ‐CD), a γ‐CD derivative with a larger cavity diameter than HP‐β‐CD, fine‐tunes UC solubilization by forming a distinct UC inclusion mode from HP‐β‐CD and restores cholesterol balance in cells and murine models of NPC more safely than HP‐β‐CD.^18^, ^19^, ^20^ We further reported the safety advantages and favourable physicochemical properties of chemically pure, mono‐branched CD derivatives with α‐1,6‐linked maltosyl derivatives over HP‐β‐CD in NPC experimental models.^21^, ^22^, ^23^ In another approach, we synthesized several drug delivery system‐based derivatives targeting affected organs to improve the bioavailability of highly excretable CDs.^24^, ^25^ Over the past two decades, clinical outcome data of HP‐β‐CD therapy for NPC and preclinical evidence for the therapeutic potential of alternative CD derivatives have been accumulating. However, clinical translation has been hindered by the limited knowledge of the biophysical properties of CDs that affect their functions.

Here, we characterized the properties of CDs that modulate their function in UC flux in the treatment of NPC. We found that the function of CDs in cellular UC flux is affected by cavity size‐dependent stoichiometry and substituent‐associated stability of CD–UC complexation. We found that the ability of CD derivatives to form 1:1 and 2:1 complexes with UC were correlated with their ability to normalize intracellular cholesterol trafficking and with their cytotoxicity associated with cellular UC removal, respectively, in a cell culture model of NPC, suggesting that these complexes predominantly serve as a shuttle and sink, respectively. We also identified that CD derivatives with a higher ability to form a 1:1 complex were more effective in extending lifespan in a mouse model of NPC when administered intracerebroventricularly. Notably, subcutaneous injection of γ‐CD derivatives caused less systemic and auditory toxicity compared with β‐CD derivatives, whereas intracerebroventricular administration induced hearing loss for all CD derivatives tested, except for a representative α‐CD derivative that had no therapeutic effectiveness probably because of negligible UC solubilization. These results suggest that the shuttle and sink model of CD function may be useful for the development of strategies for modulating UC flux for NPC treatment. Furthermore, our findings should guide further studies on the structure–activity relationship of CDs for the treatment of NPC.

## 2 METHODS

### 2.1 Reagents

In this study, 2‐hydroxypropyl‐α‐CD (HP‐α‐CD, DS: 3.3−5), HP‐β‐CD (DS: 4.4−4.6), 2‐hydroxybutyl‐β‐CD (HB‐β‐CD, DS: 4.1), HP‐γ‐CD (DS: 4.6) and 2‐hydroxybutyl‐γ‐CD (HB‐γ‐CD, DS: 3.5) were kindly donated by Nihon Shokuhin Kako Co., Ltd. In addition, mono‐6‐*O*‐α‐glucosyl‐β‐CD (G1‐β‐CD, DS: 1), mono‐6‐*O*‐α‐maltosyl‐β‐CD (G2‐β‐CD, DS: 1), mono‐6‐*O*‐α‐glucosyl‐γ‐CD (G1‐γ‐CD, DS: 1) and mono‐6‐*O*‐α‐maltosyl‐γ‐CD (G2‐γ‐CD, DS: 1) were generously supplied by Ensuiko Sugar Refining Co., Ltd. All other reagents and solvents were of reagent grade. Deionized and distilled water was used throughout the study.

### 2.2 UC solubility analysis

An excess of UC (10 mg) was added to each concentration of CD derivative in distilled water and shaken at 37°C for 3 h at 180 rpm. After equilibrium was attained (Figure S1), an aliquot was filtered through a Millex‐HP PES 0.45‐μm filter (Merck Millipore Ltd.). The filtrate was mixed and shaken for 10 min with an appropriate volume of chloroform/methanol (2:1, v/v). After centrifugation (1500 × *g*, 10 min, 4°C), the chloroform phase was recovered and evaporated. The residue was dissolved in a solvent consisting of 2‐propanol, polyoxyethylene alkyl ether and polyoxyethylene lauryl ether (87:10:3), and the concentration of UC was measured with a Determiner L FC kit (Kyowa Hakko Kirin Co., Ltd.) on a microplate reader (Tecan Group, Ltd.). Three replicate samples were performed. The binding constants of the 1:1 and the 2:1 CD:UC complexes were calculated according to a previously established method.^26^

### 2.3 Molecular docking simulation

The 3D structure file for UC was obtained from PubChem (CID 5997). The structure of each CD derivative was constructed using Avogadro software (ver. 1.2.0)^27^ on the basis of the 3D structures of native CDs that were retrieved from the Protein Data Bank (PDB ID: 4FEM for α‐CD and 1D3C for γ‐CD) (rcsb.org) and the Cambridge Structural Database (Ref. code: BCDEXD03 for β‐CD).^28^, ^29^ Based on previous reports,^30^, ^31^ the structures of hydroxyalkylated CD derivatives were prepared by assuming that four 2‐hydroxyl groups of glucopyranose units were substituted by hydroxyalkyl groups as equally spaced as possible, in accordance with the DS of hydroxyalkylated CD reagents. In brief, every other 2‐hydroxyl group is hydroxyalkylated in γ‐CD derivatives, while there is a single place where the substituted 2‐hydroxyl groups are adjacent to each other in β‐CD derivatives. The molecular geometry of UC and CD derivatives were fed into GAMESS (ver. 2019 R1)^32^ for geometry optimization using the semiempirical quantum chemistry method AM1, and no imaginary frequencies were obtained. Molecular docking to obtain 1:1 complexes of UC with CD derivatives was then performed with 300 runs using the Lamarckian genetic algorithm in AutoDock 4.2.^33^ For predicting the probable binding conformation of the 2:1 complexes of β‐CD derivatives with UC, the AutoDock program is unable to simultaneously carry out the global optimization for three molecules. Therefore, we prepared the lowest‐energy dimers of β‐CD derivatives by conducting a docking simulation between two β‐CD derivatives within the grid map restricted to the direction of the CD cavity. In our previous system using a different analytical method, we showed four dimerized forms of β‐CD derivatives: facing secondary portals (head to head, HH), facing primary and secondary portals (head to tail, HT; and tail to head, TH), and facing primary portals (tail to tail, TT).^19^ In the present system, only two dominant binding modes of dimers of β‐CD derivatives, HH and TT, were obtained through 100 docking calculations. Then, the lowest‐energy conformations of the 2:1 complexes were estimated through 100 docking runs with UC by treating the CD dimer as a rigid single receptor.

### 2.4 Cell culture

WT and *Npc1*‐null Chinese hamster ovary cells developed previously^34^ were used. The cells were grown in a culture medium consisting of a 1:1 mixture of Dulbecco's modified Eagle's medium (DMEM) and F‐12 (Life Technologies) supplemented with 10% fetal bovine serum (FBS; Thermo Fisher Scientific, Inc.). The cells were maintained at 37°C in a saturated humidity atmosphere of 95% air and 5% CO_2_.

### 2.5 Measurement of intracellular cholesterol

The measurement of intracellular cholesterol levels was performed in accordance with our previously developed method^35^ with minor modification. Cells were pre‐incubated for 24 h and then exposed to a medium containing CD derivatives for 24 h. Thereafter, the cells were lysed, and aliquots were taken for cholesterol and protein measurements. Cholesterol was extracted from the cell lysate using chloroform, 2‐propanol and NP‐40 substitute (7:11:0.1), and the solution was then centrifuged (15 000 × *g*, 10 min, 4°C). The chloroform layer was evaporated, and the residues were dissolved in a solvent consisting of 2‐propanol, polyoxyethylene alkyl ether and polyoxyethylene lauryl ether (87:10:3). The dissolved samples were divided into two portions—one was incubated with esterase to measure total cholesterol (TC), and the other was incubated without esterase to measure UC. The cholesterol content was measured with a Determiner L FC kit. The EC level was calculated by deducting the UC level from the TC level. Intracellular cholesterol content was normalized by the total protein concentration as determined with a Pierce BCA Protein Assay Kit (Thermo Fisher Scientific, Inc.).

### 2.6 Measurement of cell viability

Cell viability was measured with a water‐soluble tetrazolium salt (WST‐8) assay using a Cell‐Counting Kit‐8 (Dojindo Laboratories). The cells were incubated in 96‐well plates (1 × 10^4^ cells/well) in culture medium at 37°C for 24 h. The medium was then replaced with fresh medium containing each concentration of CD derivative for 12 h, and the cells were incubated with the WST‐8 solution for 1.5 h at 37°C. The maximum absorption of the WST‐8 formazan reagent (450 nm) was measured with a microplate reader (Tecan Group, Ltd.). Cell viability was expressed as the percentage of viable cells relative to that of the vehicle‐treated controls.

### 2.7 Measurement of UC in medium leaked from the cells by CD derivatives

The UC extracted by the various CD derivatives from the cell into the culture medium was measured. The cells were seeded in 10 cm dishes (1 × 10^5^ cells/mL), and then, 48 h later, incubated with an FBS‐free medium containing each concentration of CD derivative for 30 min. The medium was collected and centrifuged (1000 × *g*, 4°C, 10 min), and UC in the supernatants was extracted with chloroform/methanol (2:1, v/v). UC content was measured as described above.

### 2.8 Mice and administration of CD derivatives

Male and female *Npc1* homozygous mutant (BALB/cNctr‐*Npc1* ^m1N^, *Npc1* ^−/−^) mice,^36^ kindly donated by Prof. Kousaku Ohno and Dr. Katsumi Higaki, were used as a murine model of NPC. Age‐matched WT mice were used as controls. The mice were bred and housed under specific pathogen‐free conditions in the Center for Animal Resources and Development, Kumamoto University. The mice were housed in cages in a room under controlled temperature (24°C) and a 12/12‐h light/dark cycle, and provided with free access to food and water. The animal experiments were performed at the Department of Clinical Chemistry and Informatics, Graduate School of Pharmaceutical Sciences, Kumamoto University. CD derivatives were dissolved in distilled water, and the osmotic pressure was adjusted with sodium chloride to near physiological osmolality. The pH was adjusted to 7.4 using sodium hydroxide, and the solution was filtered with an Advantec DISMIC‐13CP 0.45‐μm filter (Toyo Roshi Kaisha, Ltd.). Intracerebroventricular administration was performed using stereotaxic instruments (IMPACT‐1000C and KDS 310 Plus; Muromachi Kikai Co., Ltd.) under anaesthesia (medetomidine:midazolam:butorphanol, 0.3:4.0:5.0 mg/kg, intraperitoneally). The subcutaneous injection volume was 20 mL/kg for all experimental groups. The survival times were recorded based on the day when the endpoint was reached, defined as death or the inability to consume food or drink independently.

### 2.9 Histology and immunohistochemistry

Organ samples were fixed in 4% buffered paraformaldehyde immediately after collection and then embedded in paraffin. For histopathological analysis of peripheral organs, microtome sections, 3‐μm‐thick, were prepared and stained with hematoxylin and eosin (H&E). The cerebella were immunostained for calbindin, a marker of Purkinje cells (PCs). Microtome sections of 3 μm thickness were incubated at 4°C overnight with anti‐calbindin D28K antibody (N‐18, Santa Cruz Biotechnology Inc.; 1:100 dilution), and thereafter with Histofine Simple Stain MAX PO (Nichirei) and Mayer's hematoxylin. Histopathological changes were photographed and analyzed by microscopy (BioRevo BZ‐9000; Keyence Co.).

### 2.10 Evaluation of motor function

Beam walking tests were carried out as previously described.^23^ In brief, each mouse was placed on a beam (6 mm wide, 1.1 m long and 50 cm high), and the fault rate of hindlimb steps, speed while crossing the beam and performance score were tabulated as an averaged value from two trials. From hindlimb observations, performance score was assessed in a blinded manner based on an eight‐point scale, as follows: 0, the mouse could not balance on the beam (<5 s); 1, the mouse remained on the beam for >5 s but could not cross the beam; 2, the mouse could balance on the beam but not traverse it; 3, the mouse traversed the beam with 100% foot slips or with the affected limb extended and not reaching the surface of the beam; 4, the mouse traversed the beam with ≥75% but <100% foot slips; 5, the mouse traversed the beam with ≥50% but <75% foot slips; 6, the mouse traversed the beam with <50% foot slips; and 7, the mouse traversed the beam with two or fewer foot slips.

### 2.11 Auditory brainstem response

Auditory thresholds were measured using auditory brainstem response (ABR) System 3 (Tucker‐Davis Technologies). The animals were anaesthetized by intraperitoneal administration of xylazine and ketamine‐HCl in saline. Electrodes were placed beneath the pinna of the treated ear and at the vertex just below the surface of the skin, and the ground electrode was then placed under the contralateral ear. An average of 512 sweeps was calculated at 4, 8, 12, 20 and 32 kHz. The stimulus levels near the threshold were varied in 5‐dB steps, and the threshold was defined as the lowest level at which waves in the ABR could be clearly detected by visual inspection.

### 2.12 Statistics

Statistical analyses were performed with GraphPad Prism ver. 9.5.0 (GraphPad Software). Multiple comparisons were performed to assess statistical significance. When uniform variance of the results was identified by Bartlett's analysis (*p* < .05), one‐way analysis of variance (ANOVA) was used to test for statistically significant differences. When significant differences (*p* < .05) were identified, the results were further analyzed by Dunnett's or the Tukey–Kramer multiple range test to determine the significance of differences between the groups. Where uniform variance of the results was not identified, non‐parametric multiple comparisons were performed—after confirming significant differences (*p* < .05) using Kruskal–Wallis analysis, the differences were then examined by applying Dunn's multiple comparison test. Two‐way ANOVA was performed to evaluate statistical significance. When significant differences (*p*  < .05) were identified, the data were further analyzed using Dunnett's multiple comparison test. Correlation analysis was performed using the Pearson correlation coefficient. Survival data were analyzed using the Kaplan–Meier method, and the log‐rank test was used to examine statistical significance.

## 3 RESULTS

### 3.1 Solubility and stoichiometric analyses of CD derivatives with UC

**FIGURE 1:**
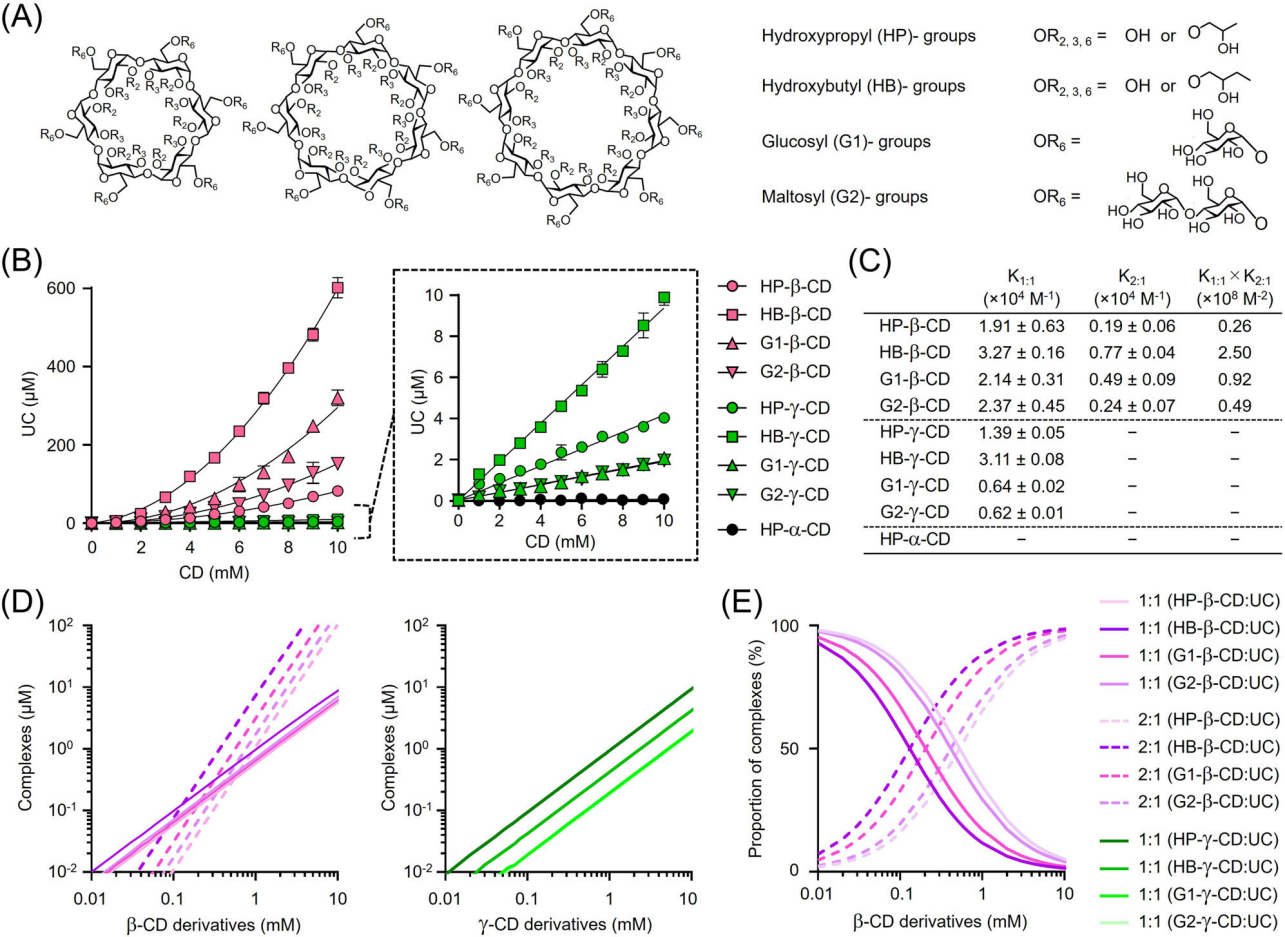
Solubility and stoichiometric studies of UC with CD derivatives. (A) Chemical structures of α‐, β‐ and γ‐CD derivatives with four different substituents—hydroxypropyl (HP), hydroxybutyl (HB), glucosyl (G1) and maltosyl (G2). The HP and HB groups are randomly substituted onto the 2, 3 and 6‐hydroxyl groups of CD and the G1 and G2 groups are solely substituted onto the 6‐hydroxyl group of CD. (B) UC solubilization profiles of CD derivatives in aqueous solution. The solubility curve of all CDs is shown in the left panel and that of γ‐CD derivatives and HP‐α‐CD are magnified in the right panel. Data represent the mean ± SD, *n* = 20 for UC solubility without CDs, *n* = 3−6 for with CDs. (C) Binding constants of 1:1 and 2:1 CD:UC complexes (K_1:1_ and K_2:1_, respectively) with standard errors calculated from the UC solubility curve by non‐linear least squares regression. (D and E) Estimated concentrations (D) and proportions (E) of 1:1 and 2:1 CD:UC complexes as a function of CD concentration from binding constants. Solid and dashed lines represent the 1:1 and 2:1 complexes, respectively.

We previously reported the differential solubility and stoichiometry profiles of HP‐β‐CD and HP‐γ‐CD with UC.^20^ Here, we assessed the profiles of nine CD derivatives—HP‐β‐CD, HB‐β‐CD, G1‐β‐CD, G2‐β‐CD, HP‐γ‐CD, HB‐γ‐CD, G1‐γ‐CD, G2‐γ‐CD and HP‐α‐CD (Figure 1A). The UC solubility diagram in the presence of CD derivatives is shown in Figure 1B. As a function of the concentration of the γ‐CD derivatives in aqueous solution, the solubility of UC increased linearly, indicating the formation of a soluble complex of the γ‐CD derivatives with UC at a 1:1 molar ratio (Figure 1B, magnified panel). In contrast, with increasing β‐CD derivatives concentration, the solubility enhancement of UC exhibited an upward deviation from linearity, suggesting the formation of higher‐order soluble complexes (Figure 1B, left panel). Assuming the sequential formation of 1:1 and 2:1 complexes of β‐CD derivatives with UC, subsequent analyses were performed. Contrary to the previous report,^37^ the UC solubilization with HP‐α‐CD, a representative α‐CD derivative, was even lower than that of γ‐CD derivatives and was negligible. We next calculated the binding constants, K_1:1_ and K_2:1_ with standard errors, for 1:1 and 2:1 complex formation, respectively (Figure 1C), and then estimated and plotted the concentrations and proportions of these complexes in aqueous solution as a function of each CD concentration (Figure 1D,E). The concentrations of the 1:1 CD:UC complexes are dependent on the K_1:1_ value, and therefore, the ability of each CD derivative to form a 1:1 complex is reflected in this value. By contrast, the concentrations of the 2:1 complexes of β‐CD derivatives are dependent on the product value of K_1:1_ and K_2:1_, and the intersection point where the ratio of the 2:1 complex predominates shifted toward the lower CD concentration, in proportion to the UC‐solubilizing capacity of β‐CD derivatives (Figure 1E).

### 3.2 Molecular modeling of 1:1 UC inclusion complexes of the CD derivatives

**FIGURE 2:**
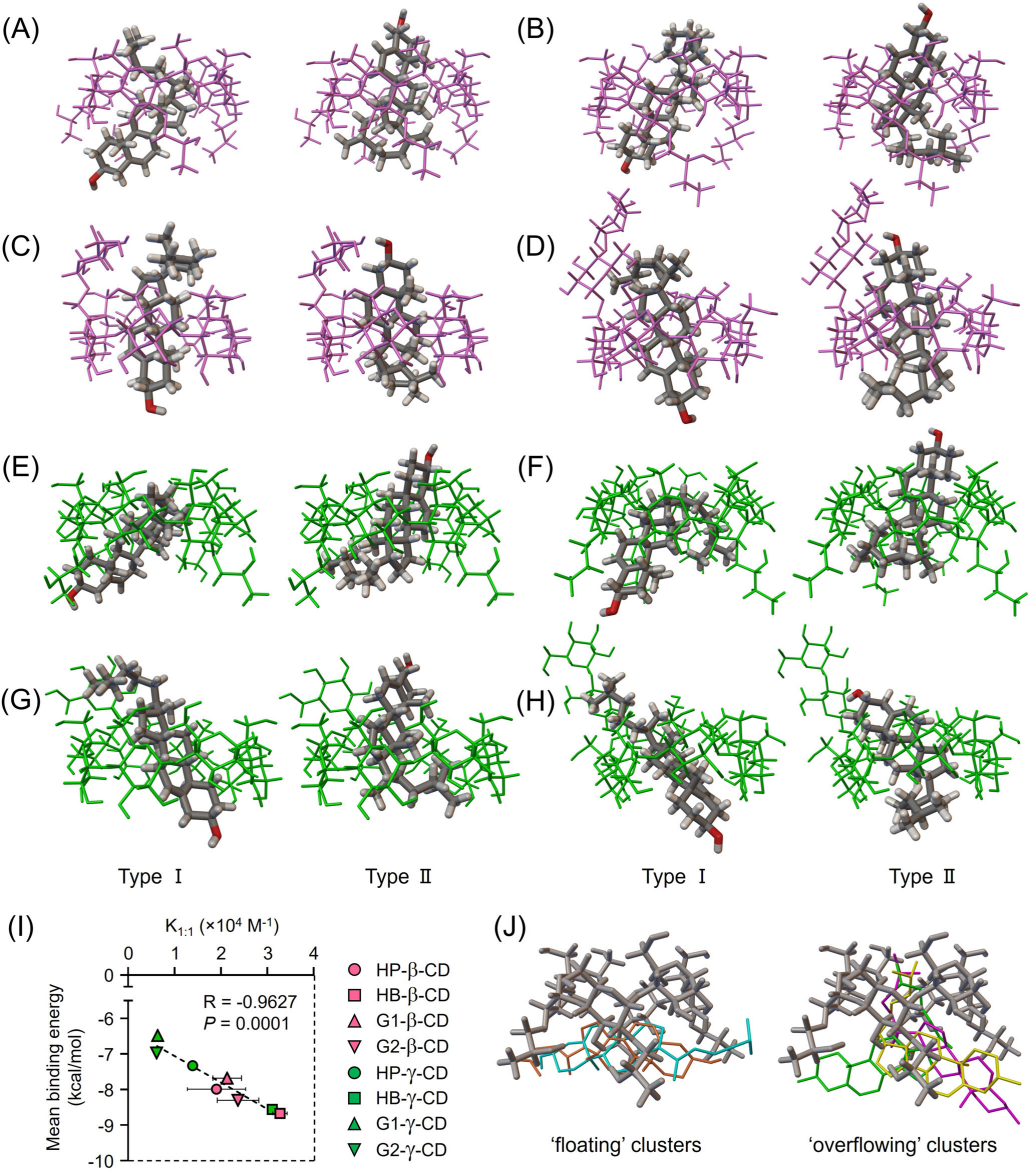
Probable binding conformation of the predominant 1:1 UC inclusion modes of CD derivatives. (A–H) The lowest UC binding energy conformations for β‐CD derivatives (A, HP‐β‐CD; B, HB‐β‐CD; C, G1‐β‐CD; D, G2‐β‐CD) and γ‐CD derivatives (E, HP‐γ‐CD; F, HB‐γ‐CD; G, G1‐γ‐CD; H, G2‐γ‐CD) in the highest populated clusters through 300 docking runs. The hydroxyl terminus of the UC molecule is directed toward the 2 and 3‐hydroxyl groups of the CD molecule in Type I (left) and is directed towards the 6‐hydroxyl group of the CD molecule in Type II (right). The oxygen atom in the UC molecule is coloured red. (I) Correlation analysis between mean binding energy of 300 runs with AutoDock and binding constants of 1:1 CD:UC complexes (K_1:1_) estimated in Figure 1. (J) The lowest binding energy conformations of the two types of clusters predicted between HP‐α‐CD and UC are depicted with different colours. UC remained unbound and floating in the vicinity of the secondary face of HP‐α‐CD in ‘floating’ clusters (left), whereas its dimethyl terminus was embedded within the cavity of HP‐α‐CD and the steroid skeleton protruded outside the cavity in ‘overflowing’ clusters (right).

To verify that γ‐CD derivatives solubilize UC by forming an inclusion complex, we used AutoDock software to predict the probable binding conformation of UC inclusion complexes by comparing with β‐CD derivatives and a representative α‐CD derivative. Through 300 docking runs, we obtained two dominant binding modes of the 1:1 CD:UC complex: the hydroxyl terminus of the UC molecule is directed toward the secondary face of the CD molecule for Type I; and the hydroxyl terminus is directed toward the primary face of the CD molecule for Type II (Figure 2A–H). The lowest binding energies for the two dominant binding conformations and the mean binding energy of 300 runs, expressed in kcal/mol, were as follows: HP‐β‐CD (Type I: −8.79, Type II: −8.29, mean: −7.99); HB‐β‐CD (Type I: −9.13, Type II: −8.76, mean: −8.67); G1‐β‐CD (Type I: −7.42, Type II: −8.08, mean: −7.68); G2‐β‐CD (Type I: −8.56, Type II: −8.59, mean: −8.30); HP‐γ‐CD (Type I: −7.75, Type II: −7.08, mean: −7.33); HB‐γ‐CD (Type I: −9.24, Type II: −8.53, mean: −8.56); G1‐γ‐CD (Type I: −6.59, Type II: −6.90, mean: −6.48); G2‐γ‐CD (Type I: −7.76, Type II: −7.47, mean: −6.97). Regardless of the specific CD derivative and binding mode, the CD ring tended to surround the steroid skeleton, suggesting the formation of a soluble inclusion complex. We also found that the mean binding energies of the 1:1 CD:UC complexes were significantly correlated with their binding constants, estimated in Figure 1. In general, stability constants determined from solubility curves have large experimental errors. However, this correlation showed that the experimental results are in line with the predictive findings, suggesting the validity of the individual experimental procedures and results (Figure 2I). Applying this method, we confirmed the inability of HP‐α‐CD, a representative α‐CD derivative, to form a soluble inclusion complex with UC. The lowest binding energy conformations of clusters obtained from 300 runs are depicted with different colours (Figure 2J). Consistent with the previous report of native α‐CD,^38^ UC either remained unbound and floating in the vicinity of the larger opening of HP‐α‐CD or its hydrophobic tail, with the dimethyl terminus embedded within the cavity of HP‐α‐CD and the steroid skeleton protruding outside the cavity, as shown in the ‘floating’ (left) and ‘overflowing’ (right) clusters, respectively. Taken together, these data suggest that γ‐CD derivatives as well as β‐CD derivatives solubilize UC by accommodating across a relatively broad region of the UC molecule, in contrast to α‐CD derivatives.

To elucidate the effect of DS on the stability of complexes of CD derivatives with UC, the binding free energies of native and mono‐substituted CDs of these corresponding CD derivatives were estimated using AutoDock (Figure S2). Compared with native β‐ and γ‐CDs, the mean binding energies of single‐hydroxyalkylated or mono‐branched β‐ and γ‐CDs were slightly low, respectively. In each hydroxyalkylated CD, the binding energies were decreased in inverse proportion to their DS. These results suggest that the type of substituent and its DS contribute to the complexation ability of CD with cholesterol.

### 3.3 Molecular modeling of 2:1 UC inclusion complexes of the β‐CD derivatives

**FIGURE 3:**
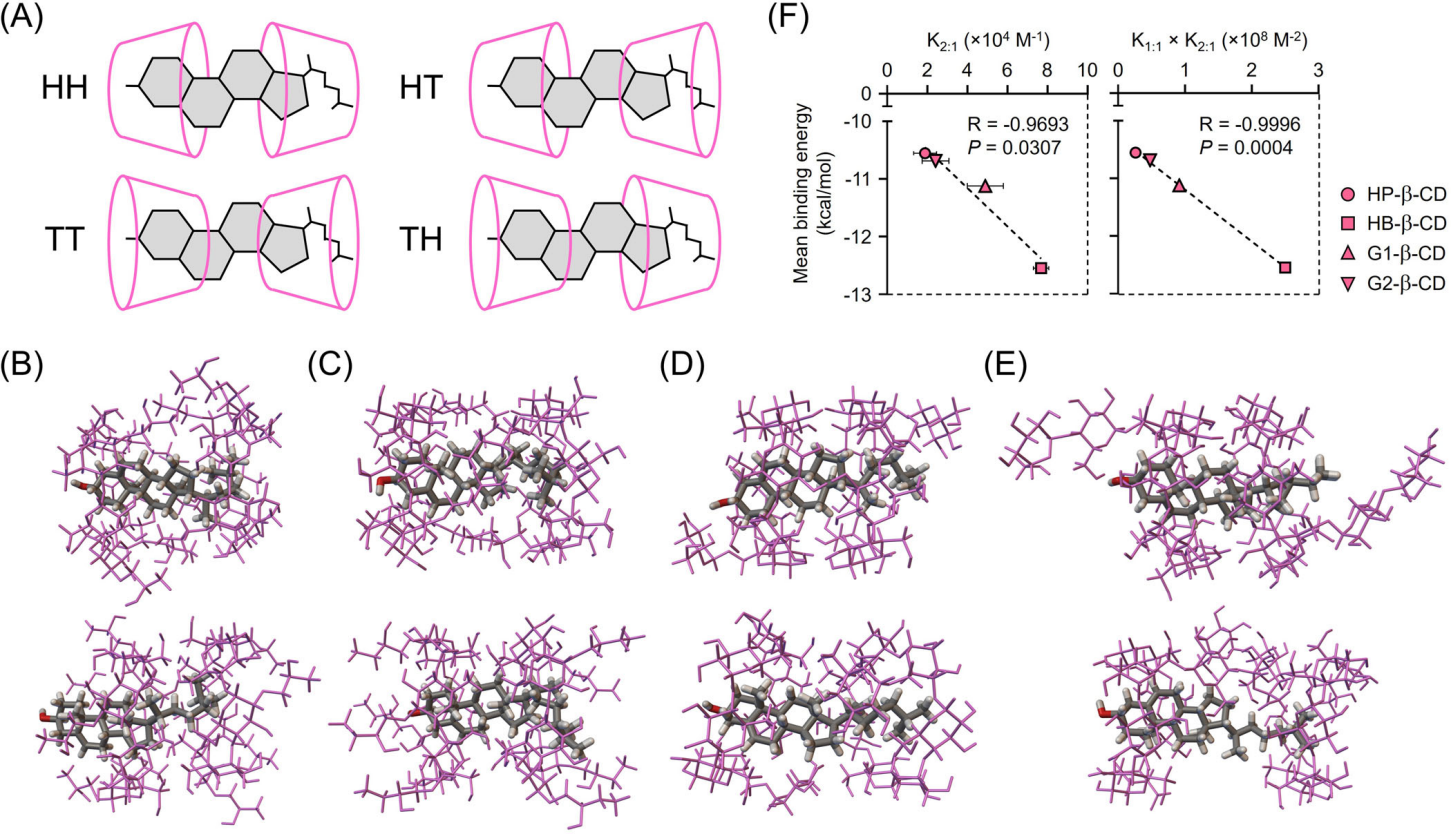
Probable binding conformation of the predominant 2:1 UC inclusion modes of β‐CD derivatives. (A) Four conceivable variations of the 2:1 binding modes of the β‐CDs and UC: HH, TT, TH, and HT. (B–E) The lowest UC binding energy conformations for dimers of β‐CD derivatives (B, HP‐β‐CD; C, HB‐β‐CD; D, G1‐β‐CD; E, G2‐β‐CD) in the highest populated clusters through 100 docking runs. The upper and lower panels show HH and TT orientation types, respectively. No HT or TH conformations were obtained in this system. The oxygen atom in the UC molecule is coloured red. (F) Correlation analysis of mean binding energy of 2:1 complexes with binding constants of 2:1 CD:UC complexes (K_2:1_) and the product of binding constants of 1:1 and 2:1 CD:UC complexes (K_1:1_ × K_2:1_) estimated in Figure 1.

We next predict the probable binding conformation of the 2:1 UC inclusion complex with β‐CD derivatives by AutoDock. In our previous study that used a different in silico molecular simulation from that applied in this study,^19^ four types of mutual orientation were conceivable between two HP‐β‐CDs and UC: HH, HT, TH, and TT (Figure 3A). The presented AutoDock analysis showed that HH and TT conformations for each β‐CD derivative and no HT or TH were obtained (Figure 3B–E). The lowest binding energies for these two binding modes and the mean binding energy, expressed in kcal/mol, were as follows: HP‐β‐CD (HH: −12.78, TT: −10.73, mean: −10.55); HB‐β‐CD (HH: −13.25, TT: −13.84, mean: −12.55); G1‐β‐CD (HH: −12.22, TT: −11.12, mean: −11.12); and G2‐β‐CD (HH: −13.08, TT: −10.73, mean: −10.69). The mean binding energies of all conformations of 2:1 complexes were significantly correlated with the K_2:1_ and K_1:1_ × K_2:1_, estimated in Figure 1, which represent the experimental values of binding affinity and the function of 2:1 complex concentration in aqueous solution at a given CD concentration, respectively (Figure 3F). Although the stability constants generally contain experimental errors, as mentioned in Figure 2, and the docking analysis could not be predictive for all four conformations, the correlation of these parameters suggests that each experimental method potentially provided reasonable results of the 2:1 complex stability.

### 3.4 Effect of CD derivatives on abnormal intracellular cholesterol trafficking in NPC model cells

**FIGURE 4:**
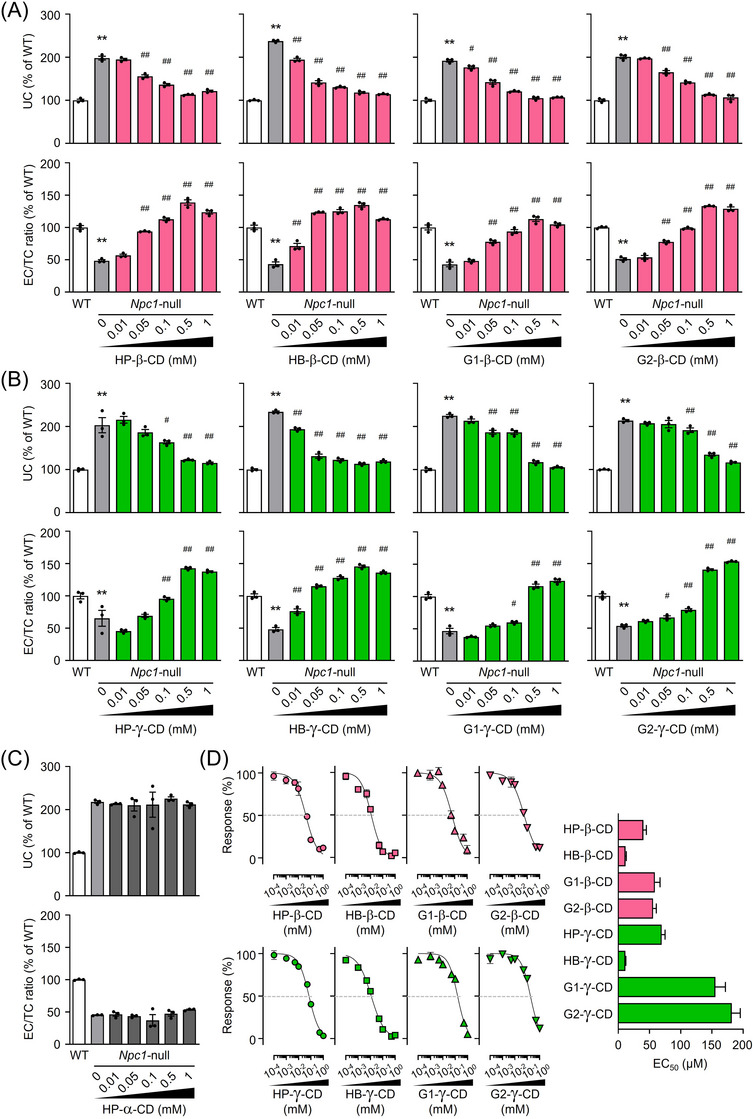
The effect of CD derivatives on the perturbed intracellular cholesterol trafficking in NPC model cells. (A–C) Concentration‐dependent effects of β‐CD derivatives (A), γ‐CD derivatives (B) and HP‐α‐CD (C) on intracellular UC levels (upper panels) and EC/TC ratio (lower panels) in *Npc1*‐null cells. (D) Concentration–response curves and EC_50_ values with standard errors of β‐ and γ‐CD derivatives on the reduction of UC levels accumulated in *Npc1*‐null cells. The UC levels correspond to the percentage of UC in cells that were normalized (the vehicle‐treated *Npc1*‐null cells and that of WT cells were used as 100% and 0% control, respectively). Data represent the mean ± SEM, *n* = 3. ^**^ *p* < .01 vs. vehicle‐treated WT cells; ^#^ *p* < .05, ^##^ *p* < .01 versus vehicle‐treated *Npc1*‐null cells.

Next, we evaluated the potential of nine CD derivatives to ameliorate the disturbed cholesterol trafficking in NPC by measuring cholesterol balance in *Npc1* gene‐trapped Chinese hamster ovary (*Npc1*‐null) cells. *Npc1*‐null cells exhibited about twice the intracellular UC levels and about half the molar ratio of EC/TC compared with wild‐type Chinese hamster ovary (WT) cells (Figure 4A−C). These accumulations of UC decreased with all β‐ and γ‐CD derivatives tested in a concentration‐dependent manner, reaching near WT levels at 1 mM (Figure 4A and B). The EC/TC ratio was significantly restored by β‐ and γ‐CD derivatives above 0.1 mM. Notably, these effects were significantly diminished when using HP‐α‐CD (Figure 4C). We and other groups previously demonstrated for several CD derivatives that their cellular uptake via fluid‐phase endocytosis is responsible for intracellular UC reduction in NPC model cells, by suppressing this process with low‐temperature incubation and pharmacological strategies.^21^, ^39^ To test this for CD derivatives used in the present study, we compared their effects within physiological and cooling conditions (Figure S3). Their normalizing effects for intracellular cholesterol balance in *Npc1*‐null cells were markedly diminished at a low temperature, suggesting that the pathway of cellular internalization for these CD derivatives is similar to the previous results. We then focused on the UC‐lowering effects of these CD derivatives by examining lower concentrations, up to 1 mM (Figure 4D). The 50 % effective concentration (EC_50_) values of each CD were as follows: HB‐β‐CD (11.6 μM) ≈ HB‐γ‐CD (11.5 μM) > > HP‐β‐CD (40.3 μM) > G2‐β‐CD (56.0 μM) ≈ G1‐β‐CD (59.6 μM) > HP‐γ‐CD (69.6 μM) > > G1‐γ‐CD (155.6 μM) > G2‐γ‐CD (181.8 μM).

### 3.5 Cytotoxicity and cellular UC efflux by CD derivatives

**FIGURE 5:**
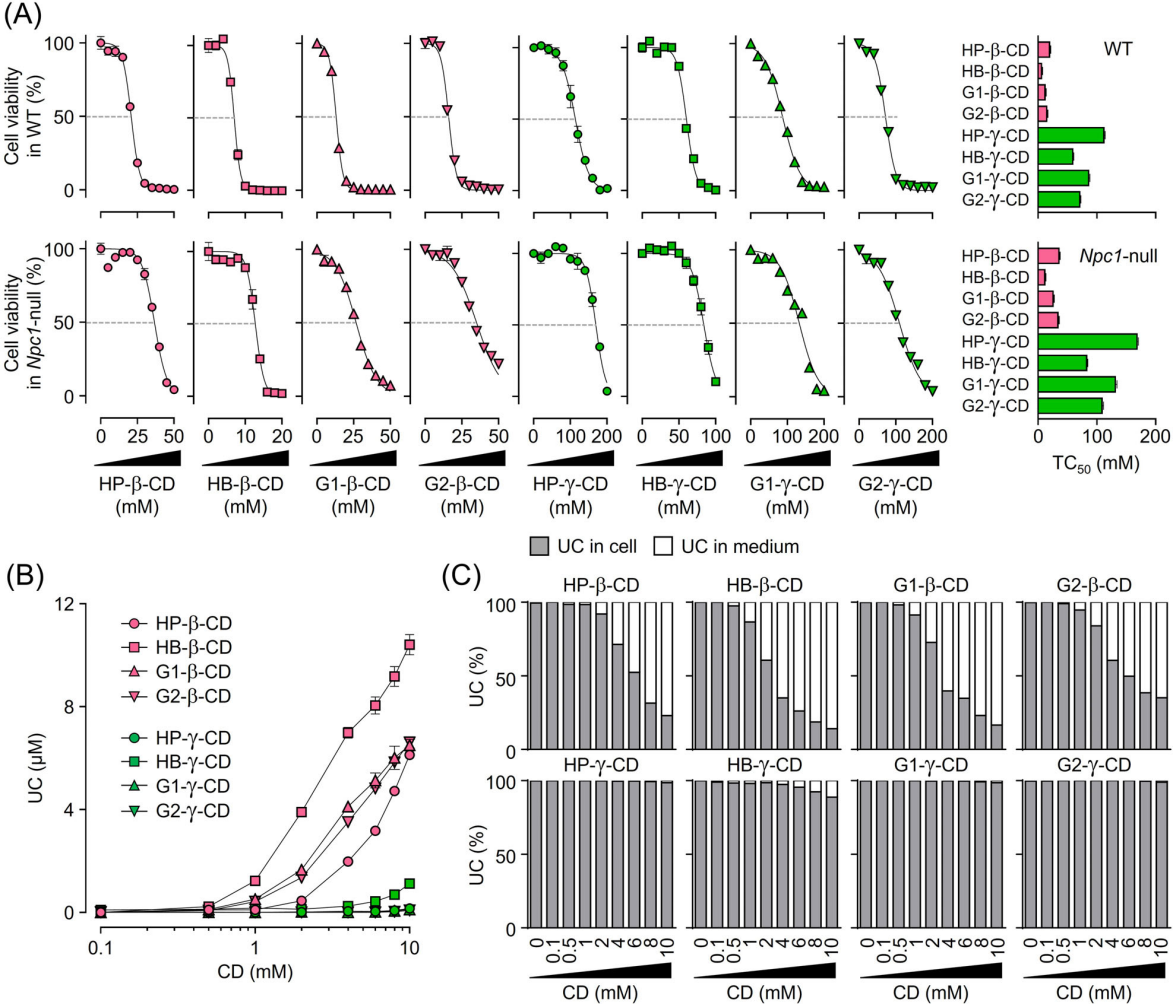
Comparisons of the cytotoxicity and UC‐extracting ability of CD derivatives in normal and disease model cells. (A) Concentration–response curves and TC_50_ values with standard errors for β‐ and γ‐CD derivatives on cell viability in WT (top panels) and *Npc1*‐null (bottom panels) cells. (B) UC leakage into the medium from WT cells following short‐term exposure to β‐ and γ‐CD derivatives of different concentrations. (C) The molar ratio of UC content in the medium (white) and the cell (grey) after brief treatment with β‐ (top panels) and γ‐CD derivatives (bottom panels). Data represent the mean ± SEM, *n* = 3−4.

Next, we evaluated and quantitatively compared the cytotoxicity of CD derivatives in both healthy and diseased Chinese hamster ovary cells. The cytotoxic effects, profiled with 50% toxic concentration (TC_50_) values, were significantly higher in β‐CD derivatives compared with γ‐CD derivatives in WT cells (Figure 5A, upper panel). A similar result was also seen in *Npc1*‐null cells (Figure 5A, lower panel). We previously reported that the cytotoxic effect of HP‐β‐CD was influenced by the presence of NPC1 protein.^40^ Consistent with this, the decreases in cell viability in *Npc1*‐null cells caused by CD derivatives were lower than those observed in WT cells (Figure 5A). Our previous study demonstrated a positive correlation between the hemolytic activity of several CDs and their ability to solubilize UC.^41^ Other researchers have proposed that CDs might function as extracellular UC reservoirs (“sinks”) at higher concentrations.^42^ To investigate the relationship between cytotoxicity and this sink capacity, we assessed the ability of CD derivatives to extract and solubilize UC from cells into the medium (Figure 5B). In parallel with UC‐solubilizing ability (Figure 1B), the efflux of cellular UC into the lipoprotein‐free medium was robustly promoted by β‐CD derivatives. The molar ratio of UC content in the medium to that in the cell suggests that leakage of cellular UC from the cell surface into the medium caused by β‐CD derivatives was significant, while that caused by γ‐CD derivatives was negligible (Figure 5C). These results suggest that β‐CD derivatives exhibit enhanced cytotoxicity by serving as extracellular UC sinks, based on their stoichiometric properties, forming higher‐order complexes distinct from γ‐CD derivatives.

### 3.6 Relationship between CD–UC complex stoichiometry and the UC‐lowering effect and cytotoxicity of CD derivatives in the NPC cell model

**FIGURE 6:**
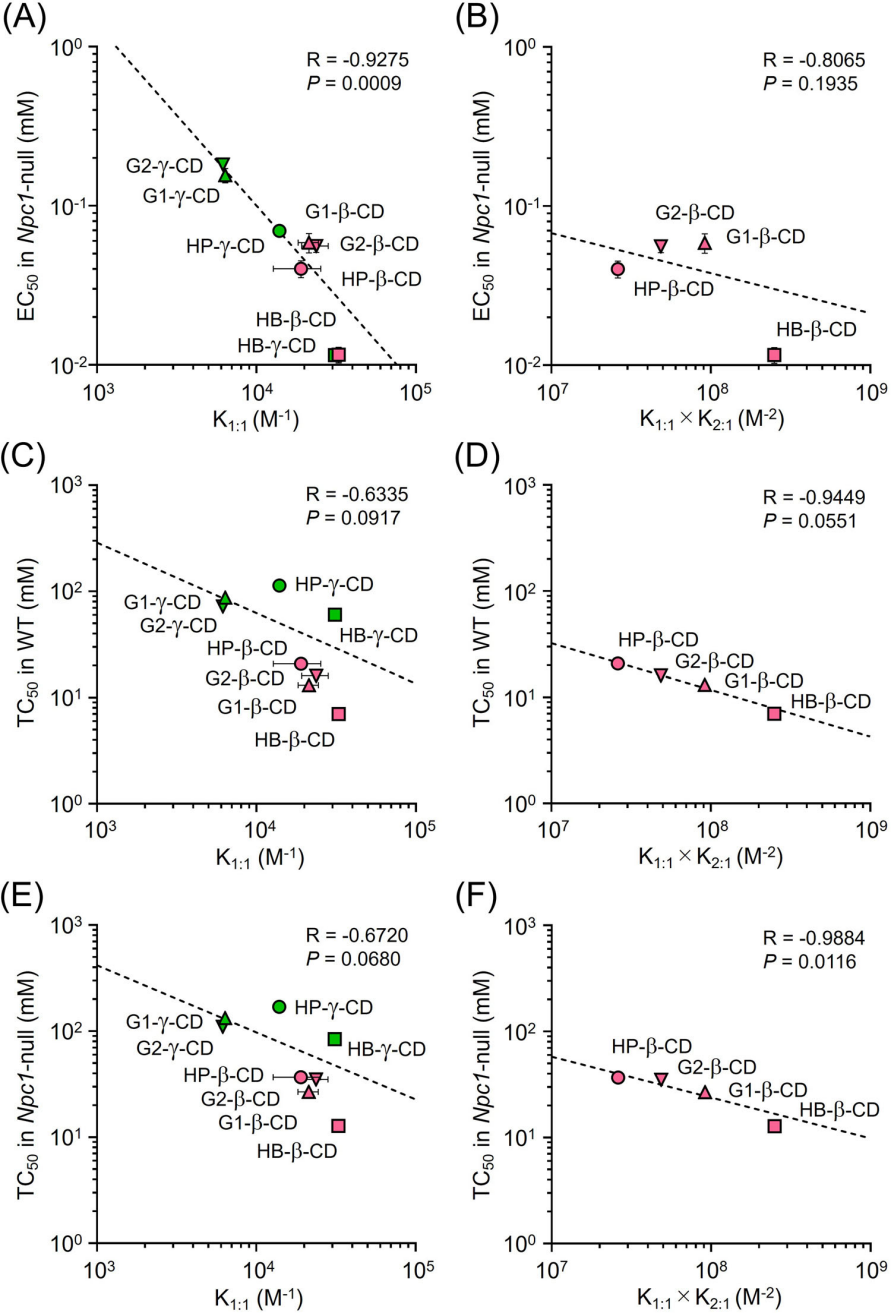
Impact of stoichiometry in CD–UC complexation on normalizing intracellular cholesterol trafficking and cytotoxicity of CD derivatives in model cells. (A and B) Scatter plots of EC_50_ values for UC accumulation in *Npc1*‐null cells and the binding constants of the 1:1 complex (A) or the product of binding constants of 1:1 and 2:1 complexes (B) for each CD derivative. (C and D) Relationships between TC_50_ values of CD derivatives for cell viability in WT cells and their binding constants of 1:1 complexes (C) or the product of binding constants of 1:1 and 2:1 complexes (D). (E and F) Relationships between TC_50_ values of CD derivatives for cell viability in *Npc1*‐null cells and their binding constants of 1:1 complexes (E) or the product of binding constants of 1:1 and 2:1 complexes (F).

We next investigated the functional role of CD–UC complex stoichiometry in cholesterol trafficking and cytotoxicity of CD derivatives in model cells. As shown in Figure 1C, the concentrations of 1:1 and 2:1 CD:UC complexes in aqueous solution at a given CD concentration were dependent on the K_1:1_ and the product of K_1:1_ and K_2:1_, respectively. The EC_50_ values of CD derivatives for UC accumulation in *Npc1*‐null cells, shown in Figure 4D, were significantly correlated with their K_1:1_ values (Figure 6A), but not with K_1:1_ × K_2:1_ values (Figure 6B). Furthermore, there were no significant correlations between TC_50_ values and K_1:1_ values of CDs in WT cells (Figure 6C). A similar result was obtained with *Npc1*‐null cells (Figure 6E). In addition, there was a trend toward a correlation of TC_50_ values with K_1:1_ × K_2:1_ values for four β‐CD derivatives in WT cells (Figure 6D). In *Npc1*‐null cells, statistical significance was obtained between these parameters (Figure 6F). Taken together, these results suggest that the ability of CD derivatives to form 1:1 and 2:1 complexes with UC affects their ability to normalize intracellular cholesterol trafficking (shuttle) and their ability to increase the cytotoxicity associated with UC extraction from the cells (sink), respectively, in model cells.

### 3.7 Therapeutic effect of intracerebroventricular administration of CD derivatives in NPC model mice

**FIGURE 7:**
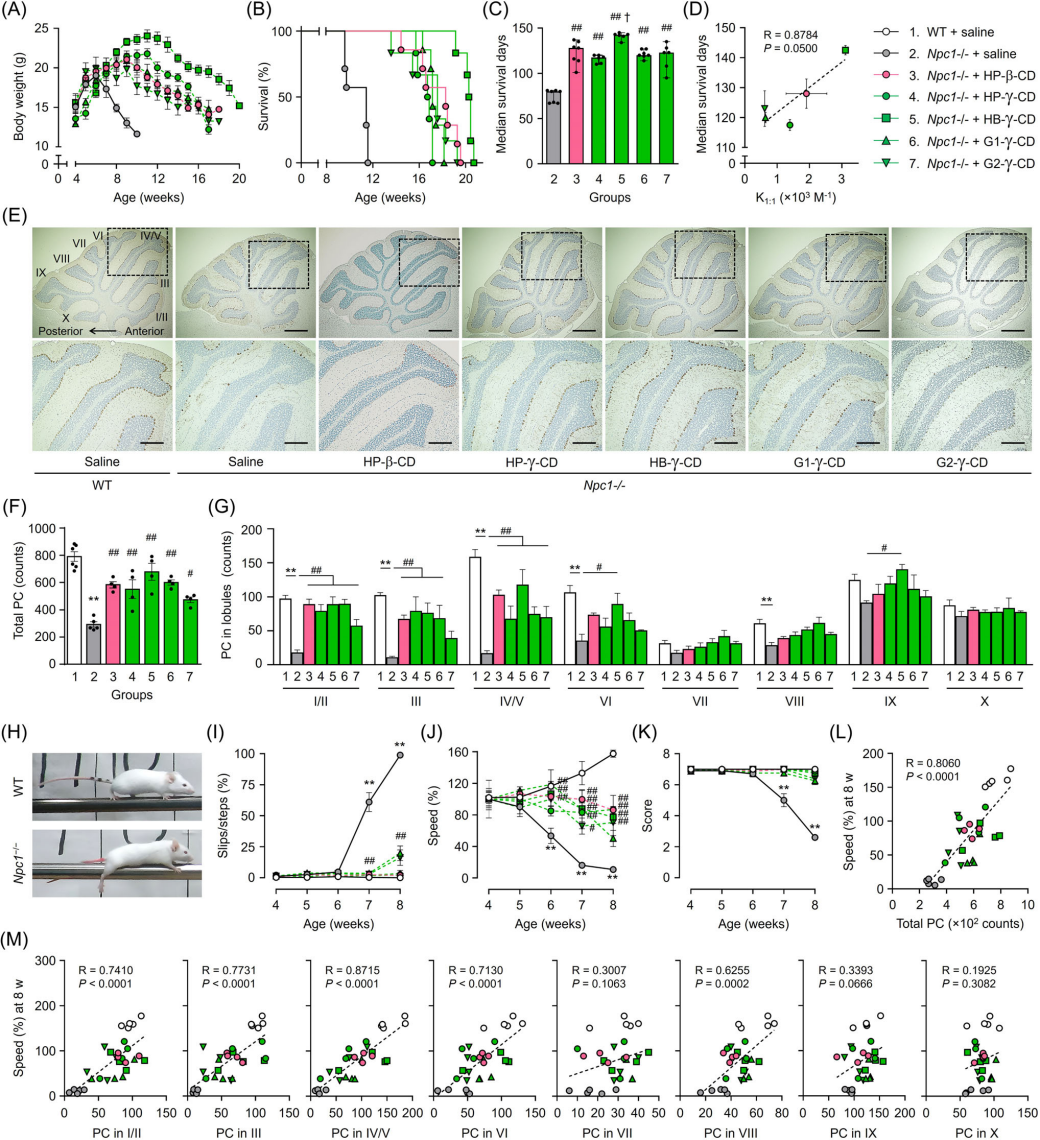
Ameliorating effect of intracerebroventricular administration of CD derivatives on NPC‐related manifestations in model mice. (A–C) Weekly weight change of mice (A); Kaplan–Meier survival curve (B); and median lifespan (C) of the mice (*n* = 6−7). (D) Relationship between median survival days of mice and the binding constants of 1:1 complexes of CD derivatives with UC. (E) Representative images of sagittal sections cut through the cerebellar vermis consisting of lobules I to X. Scale bars: 500 μm (upper panel) and 200 μm (bottom panel). (F and G) Quantitative analysis of Purkinje cell (PC) counts in whole (F) and in each lobule (G) in sagittal cerebellar sections. (H) Walking posture of mice on the beam at 8 weeks of age. (I–K) Average values of the slip rate of hindlimb steps (I), speed (% of the value at 4 weeks of age) while walking on the beam (J), and performance score (K), quantified through weekly beam walk test. (L and M) Relationships between the average speed obtained from the latest trial before sacrifice at 8 weeks of age and PC counts in whole (L) or in each lobule (M) of the cerebellum. *n* = 4−5. The mice were divided into the following groups: 1, saline‐treated WT; 2, saline‐treated *Npc1* ^−/−^; 3, HP‐β‐CD‐treated *Npc1* ^−/−^; 4, HP‐γ‐CD‐treated *Npc1* ^−/−^; 5, HB‐γ‐CD‐treated *Npc1* ^−/−^; 6; G1‐γ‐CD‐treated *Npc1* ^−/−^; and 7, G2‐γ‐CD‐treated *Npc1* ^−/−^. Data represent the mean ± SEM. ^**^ *p* < .01 versus saline‐treated WT mice; ^#^ *p* < .05, ^##^ *p* < .01 versus saline‐treated *Npc1* ^−/−^ mice.

Despite the recent clinical reports of intrathecal HP‐β‐CD therapy for NPC,^14^, ^15^ there are no preclinical reports comparing the therapeutic effects of different CD derivatives following central administration. To address this, we intracerebroventricularly injected HP‐β‐CD and γ‐CD derivatives, which have lower cytotoxicity than β‐CD derivatives as shown in Figure 5, at a dose of 21.4 μmol/kg (approximately 30 mg/kg as HP‐β‐CD) to *Npc1* homozygous mutant (*Npc1* ^−/−^) mice once at 4 weeks of age. Body weight progressively decreased after 8 weeks of age in *Npc1* ^−/−^ mice, compared with WT mice, and this decline was mitigated by CD derivatives, especially by HB‐γ‐CD (Figure 7A). The shortened lifespan and median survival time in *Npc1* ^−/−^ mice was drastically prolonged by single intracerebroventricular administration of CD derivatives, especially HB‐γ‐CD, with a significant difference compared with HP‐β‐CD (Figure 7B,C). In addition, the K_1:1_ values of CD derivatives were significantly associated with median survival of *Npc1* ^−/−^ mice (Figure 7D). Thus, the 1:1 complexation ability of CD derivatives with UC affects therapeutic effectiveness in NPC model mice.

At 8 weeks of age, when NPC pathology progressed, the cerebella of *Npc1* ^−/−^ mice showed a marked decrease in the total number of calbindin‐positive PCs compared with WT mice (Figure 7E,F). Consistent with the previous report,^43^ this cell vulnerability across individual lobules exhibited an anterior‐to‐posterior gradient, with no appreciable loss in nodular regions (Figure 7G). Single intracerebroventricular injection of the CD derivatives significantly protected against this neurodegeneration predominantly in the anterior to central zones. Because PC dysfunction and loss underlie the cerebellar ataxia, a characteristic neuropathological feature of NPC, we performed the beam walk test to evaluate motor function weekly from 4 to 8 weeks of age. A marked increase in the rate of hindlimb missteps and slips, and significant reductions in average speed and performance score were seen in *Npc1* ^−/−^ mice traveling a beam, compared with WT mice (Figure 7H–K). These progressively worsening deficits were markedly mitigated by single intracerebroventricular administration of CD derivatives. In addition, total PC count was significantly correlated with average speed of walking on the beam during the latest trial before sacrifice (Figure 7L). This correlation was particularly pronounced in the anterior to central lobes (lobules I–VI) and lobule VIII (Figure 7M), consistent with the links between these lobules and the cerebral cortical areas involved in sensorimotor processing.^44^

### 3.8 Systemic biocompatibility of CD derivatives in mice

**FIGURE 8:**
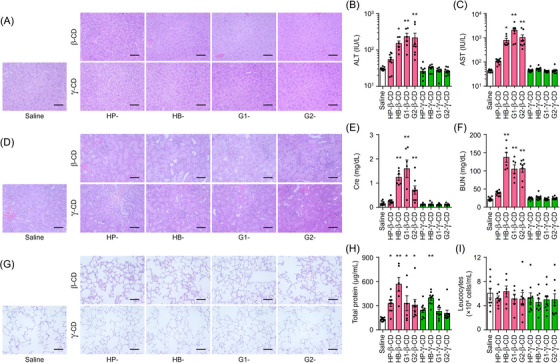
Biocompatibility evaluation of systemic administration of CD derivatives on peripheral organs of mice. (A) Representative liver histology of mice treated subcutaneously with saline, β‐CD derivatives (upper panels) and γ‐CD derivatives (lower panels). Scale bars: 100 μm. (B and C) The changes in serum alanine transaminase (ALT) (B) and aspartate transaminase (AST) (C) levels in mice. (D) Representative renal histology of mice treated subcutaneously with saline, β‐CD derivatives (upper panels) or γ‐CD derivatives (lower panels). Scale bars: 50 μm. (E and F) The changes in creatinine (E) and BUN (F) in serum. (G) Representative images of lung sections of mice treated subcutaneously with saline, β‐CD derivatives (upper panels) or γ‐CD derivatives (lower panels). Scale bars: 50 μm. (H and I) The total protein concentration (H) and leukocyte count (I) in the BALF. All tissue sections were stained with H&E. Data represent the mean ± SEM, *n* = 6−9. ^*^ *p* < .05, ^**^ *p* < .01 versus saline‐treated group.

To confirm the CD:UC stoichiometry‐related differences in the toxicity of CD derivatives observed in vitro, toxicity toward peripheral organs was evaluated in mice. We subcutaneously administered a 5.7 mmol/kg dose of the CD derivatives (approximately 8000 mg/kg of HP‐β‐CD, twice the dose shown to be effective in NPC model mice) into WT mice at 8 weeks of age, and samples were collected 24 h later. In the β‐CD derivative‐treated groups, hepatocellular necrosis and shortages in the glycogen pool were observed in liver tissues (Figure 8A). Significant increases in serum transaminase levels were also seen in these mice (Figure 8B,C). In contrast, negligible changes in hepatic histology and serum parameters were observed in γ‐CD derivative‐treated groups. Vacuolation of the tubular epithelium and elevated serum creatinine and blood urea nitrogen (BUN) were induced by β‐CD derivatives, while such changes were not seen following administration of γ‐CD derivatives (Figure 8D–F). Lung histology showed hemorrhage and thickened alveolar septa following β‐CD derivative treatment, whereas no significant changes were observed in the γ‐CD derivative‐treated groups (Figure 8G). Bronchoalveolar lavage fluid (BALF) analysis demonstrated significant increases in total protein concentration in mice treated with β‐CD derivatives or HB‐γ‐CD, indicating pulmonary vascular hyperpermeability, in contrast to the other γ‐CD derivatives (Figure 8H). There were no significant changes in total leukocyte count in the BALF among the different treatment groups (Figure 8I). Given our previous report that G2‐β‐CD was rapidly biodegraded to G1‐β‐CD by α‐glucosidase in whole blood of several experimental animals, with the rate of degradation varying among species,^45^ it should be noted that maltosylated CDs may function in vivo as its metabolite, glucosylated CDs. In summary, these in vivo findings are in line with the in vitro findings on the relationship between the complexation stoichiometry of CD derivatives with UC and cytotoxicity, revealing differences in the biocompatibility of β‐ and γ‐CD derivatives.

### 3.9 Biocompatibility of CD derivatives in auditory organs of mice

**FIGURE 9:**
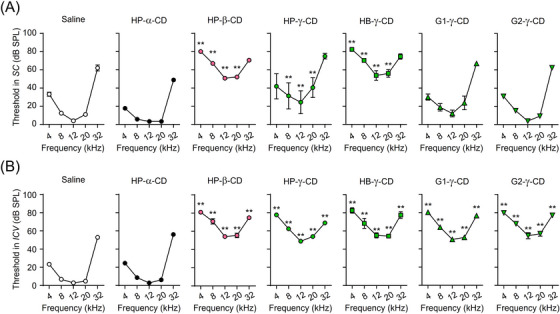
Ototoxic effect of subcutaneous or intracerebroventricular delivery of CD derivatives in mice. Hearing thresholds expressed as decibels of sound pressure level (SPL) for five frequencies were measured 1 week after a single subcutaneous (*SC*, 5.7 mmol/kg) (A) or intracerebroventricular (*ICV*, 21.4 μmol/kg) (B) administration of CD derivatives into WT mice at 8 weeks of age. Data represent the mean ± SEM, *n* = 4−6. *p < .05, **p < .01 versus saline‐treated group.

Some preclinical and clinical studies show that the administration of HP‐β‐CD induces severe auditory dysfunction in patients and animal models of NPC.^15^, ^46^ To investigate the impact of CD:UC stoichiometry on auditory function, we compared the ototoxicity of CD derivatives after a single subcutaneous or intracerebroventricular injection into mice. Subcutaneous injection of 5.7 mmol/kg HP‐β‐CD significantly increased the ABR threshold at every measured frequency, compared with saline treatment, while only a moderate increase and negligible changes were obtained following administration of HP‐γ‐CD and branched γ‐CDs, respectively (Figure 9A). In comparison, intracerebroventricular administration of a 21.4 μmol/kg dose induced a significant elevation of the threshold for all CD derivatives that showed therapeutic effectiveness in NPC model cells and mice (Figure 9B). Notably, HP‐α‐CD, a representative α‐CD derivative that is unable to accommodate the UC molecule, did not cause any change in the threshold, whether administered subcutaneously or intracerebroventricularly. Collectively, these results suggest that the UC inclusion ability of CD derivatives plays a critical role in the induction of hearing impairment.

## 4 DISCUSSION

Here, we showed that the cavity size‐dependent stoichiometry and substituent‐associated stability in the UC inclusion complex of various CD derivatives impact their therapeutic and toxicological properties in the treatment of experimental models of NPC. We revealed that the abilities of CD derivatives to form 1:1 and 2:1 complexes with UC, which are reflected by their binding constants, were significantly correlated with their ability to normalize intracellular cholesterol trafficking and with their cytotoxicity associated with UC extraction from the cells, respectively, in model cells. We further showed that these correlations are consistent with their therapeutic effectiveness and systemic biocompatibility/toxicity in model mice. However, we also identified a significant vulnerability of the auditory system to UC‐accommodating CD derivatives when administrated intracerebroventricularly. The negligible change in auditory function caused by HP‐α‐CD, a representative α‐CD derivative with no therapeutic effectiveness probably because it is incapable of solubilizing UC, suggests that ototoxicity is preventable by restricting UC accommodation by CD derivatives at the cell surface. Our current findings should further our understanding of the function of CD derivatives and facilitate the optimization of their molecular structure for the treatment of NPC.

The stability of the 1:1 and 2:1 CD:UC complexes, which were correlated with ameliorative effects on cellular pathology and cytotoxicity, respectively, is considered to be affected by the depth and asymmetry of the hydrophobic CD cavities, which are related to the physicochemical properties of the substituent, such as relative hydrophobicity and steric bulkiness, as well as its DS.^47^, ^48^, ^49^ In this study, we showed that the calculated mean binding energies of single‐hydroxyalkylated or mono‐branched β‐ and γ‐CDs were slightly lower than those of native β‐ and γ‐CDs, respectively. Furthermore, the binding energies of each hydroxyalkylated CD decreased in inverse proportion to their DS. These results, partially supported by the slight difference of stability constants in native β‐CD and HP‐β‐CD with UC,^50^ suggest that the extension and/or asymmetrization of the hydrophobic cavity of CD by substituents affect its complexation ability. Similar to the 1:1 complex, the above factors involved in substituents may contribute to the stability of the 2:1 complex as well. A previous report showed a biphasic DS‐dependent UC‐solubilizing ability of HP‐β‐CDs, with maximum solubility around DS 7.^49^ This suggests that, as the DS of hydroxyalkyl groups increases, the hydrophobic cavity of the two CD molecular shells expands while an excessive increase in DS leads to increased steric hindrance between the substituents of the two CDs. In addition, our previous result, which showed the absence of a significant cross peak between UC and the maltose of G2‐β‐CD in 2D ^1^H‐NMR analysis, suggests that the glucose and maltose moieties of branched CDs may contribute to the interaction with UC by altering the symmetry of the CD cavity, rather than through hydrophobic interaction, regardless of the order of complex.^22^ Therefore, evaluating the impact of substituents on the complex stability becomes more complicated in 2:1 complex because not only CD–UC interaction but also the interaction between two CDs, including the mutual orientation of CD rings, the distribution of substituents, and the resulting ring distortion, should be considered.

**FIGURE 10:**
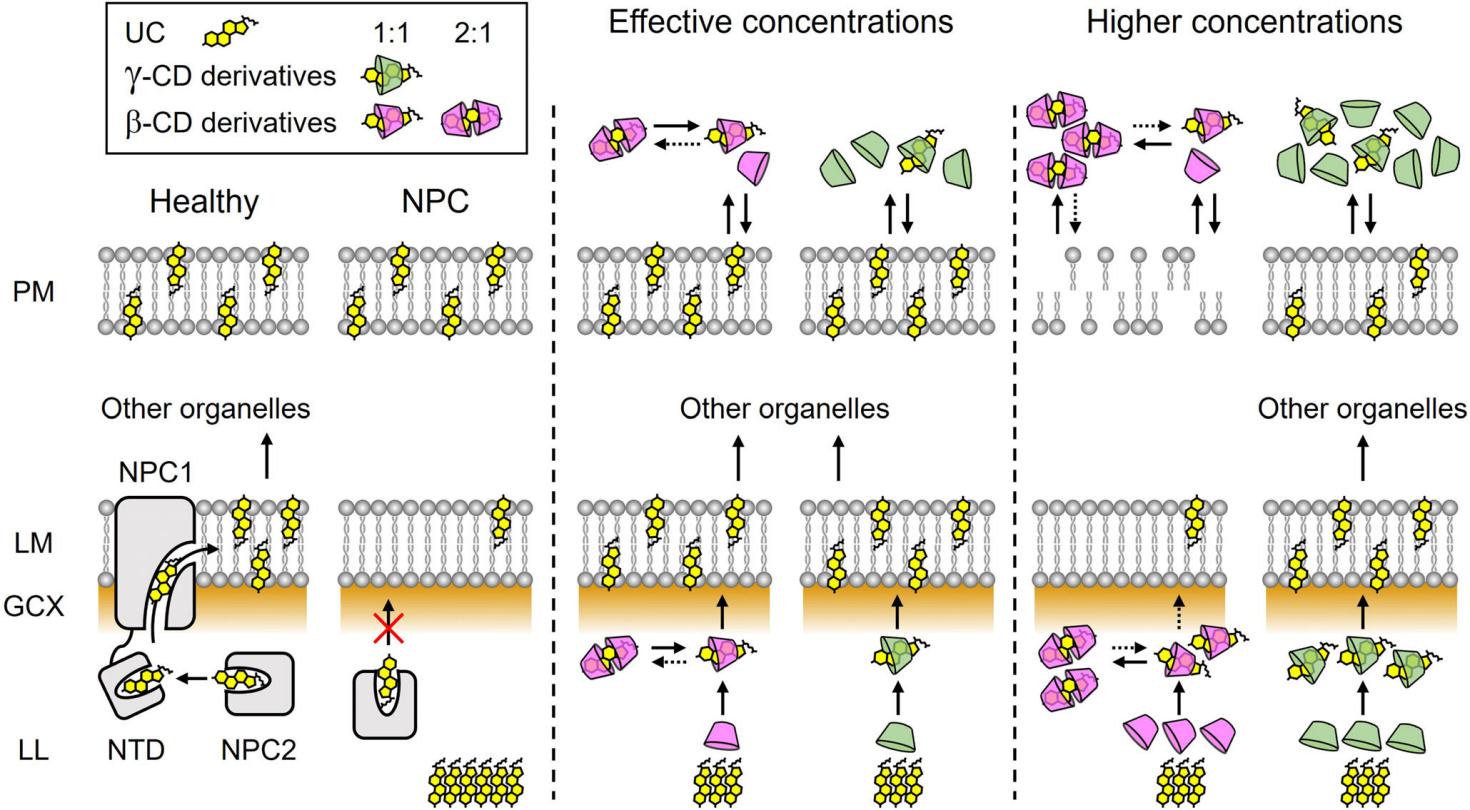
Proposed model of the regulation of cellular UC flux by CDs (applying the shuttle and sink mechanism) in NPC treatment. Left: Critical pathway of intracellular cholesterol trafficking through the cooperation of NPC proteins in normal conditions and its disturbance in disease states where NPC1, including its N‐terminal domain (NTD), is absent. Middle: At effective concentrations, β‐ and γ‐CD derivatives form a 1:1 inclusion complex with UC and act as a shuttle, thereby facilitating UC transport from the lysosomal lumen (LL) to the lysosomal membrane (LM) through the glycocalyx (GCX) barrier, by functionally substituting for NPC proteins. Right: At higher concentrations, β‐CD derivatives form a highly soluble 2:1 complex with UC, acting as a strong sink to excessively extract and solubilize UC from the plasma membrane (PM), causing cytotoxicity.

Recently, the mechanisms of lysosomal UC egress mediated by NPC1 and NPC2 proteins have become increasingly well characterized.^51^, ^52^ In brief, lysosomal UC is delivered by NPC2 to the N‐terminal domain (NTD) of NPC1 in the lysosomal lumen (LL) and is then transferred to the lysosomal membrane (LM) through the tunnel pathway connecting the NTD and the transmembrane sterol‐sensing domain in NPC1 (Figure 10, left). In the absence of NPC1, UC transport to the LM by NPC2 (around 20 kDa) alone seems to be impeded by a physical barrier called the glycocalyx (GCX), which consists of oligosaccharides on glycoproteins covering the lysosomal inner membrane.^53^ The present findings, together with previous observations,^8^, ^9^, ^20^, ^21^, ^47^, ^54^, ^55^, ^56^ suggest that the mechanisms by which intracellular cholesterol trafficking is normalized by effective concentrations of CDs are as follows: membrane‐impermeable β‐ and γ‐CD derivatives, which reach the lysosome compartment via endocytosis, form a 1:1 inclusion complex with UC. When they exhibit higher stability, they more efficiently serve as a “shuttle” to deliver UC from its accumulated site to the UC‐depleted LM under thermodynamic equilibrium, easily passing through the GCX layer as smaller molecules (1–2 kDa), functionally substituting for the NPC proteins (Figure 10, middle). Note that this mechanism, based on the correlation between the 1:1 complexation abilities of CD derivatives and their ability to normalize intracellular cholesterol trafficking, is not fully established since the rate of distribution of each CD to the lysosome is not quantified. As we and another group previously demonstrated for several CDs, their cellular uptake via fluid‐phase endocytosis is responsible for restoring the intracellular cholesterol balance in NPC model cells.^21^, ^39^ Consistent results were obtained for nine CD derivatives used in this study, suggesting similar pathways of their cellular internalization. Given that, in fluid‐phase endocytosis, the cell membrane engulfs surrounding fluid and solutes,^57^ it seems potentially reasonable to apply the CD concentration in the extracellular environment to the endocytic pathway. In addition to this shuttle function, it has been argued that CDs facilitate UC net efflux from the cell membrane and can themselves act as extracellular UC reservoirs (“sinks”).^8^, ^9^ Our present findings suggest that, with increasing CD concentration, the concentration of the 2:1 complex, in which the UC molecule is totally encapsulated by two hydrophilic molecular shells of β‐CD derivative, increases and its function as a sink becomes predominant, resulting in excessive UC removal from the plasma membrane, increased membrane fluidity, perturbed membrane integrity, and, ultimately, cell death (Figure 10, right), confirming and extending previous studies.^19^, ^41^, ^58^, ^59^

Notably, this shuttle and sink model of CD derivatives in UC flux for the treatment of NPC is not fully applicable to their impact on auditory function. The present ABR analysis revealed that ototoxicity was not caused by subcutaneous injection of several γ‐CD derivatives, compared with HP‐β‐CD, while it was induced by intracerebroventricular administration of all CD derivatives that showed therapeutic effectiveness in NPC experimental models. These differences in ototoxicity may be due to differences in the concentration of the administered CD solution (approximately 150 and 320 mM for subcutaneous and intracerebroventricular administration, respectively) as well as differences in local CD concentration in the cochlear region associated with blood–brain barrier and/or blood–labyrinth barrier permeabilities.^60^ Furthermore, our results with a representative α‐CD derivative, which has no therapeutic effectiveness probably because of negligible UC‐solubilizing ability, had no notable ototoxicity, suggesting selective vulnerability of the auditory system to UC‐chelating CD derivatives. Because CD‐induced hearing impairment is likely caused by the loss of outer hair cells, resulting from UC extraction from the lateral wall,^61^ a structural CD variant that does not bind UC at the cell surface, but only at intracellular UC accumulation sites such as lysosomes, may exhibit biocompatibility and effectiveness for the treatment of NPC.

Our findings indicate that the shuttle and sink functions of CD in UC flux are strongly impacted by complexation stoichiometry. Furthermore, they highlight the importance of strategies for modulating the molecular structure of CD derivatives to optimize therapy for NPC. Although further studies are needed to elucidate the pathogenesis of auditory dysfunction associated with outer hair cell loss induced by CDs, molecular optimization strategies may overcome these adverse effects for the treatment of NPC. In addition, modifying the molecular structure of CD raises concerns about its stability. When developing and formulating an ideal candidate compound based on this study, it will be necessary to investigate the stability, such as by accelerated temperature and freeze‐thaw tests.

## CONFLICT OF INTEREST STATEMENT

The authors declare no conflict of interest.

## Supporting information



Supporting Information

## Data Availability

The data that support the findings of this study are available from the corresponding author upon reasonable request.
