# Supplementary material for: Different solubilizing ability of cyclodextrin derivatives for cholesterol in Niemann–Pick disease type C treatment
Source: Clin Transl Med. 2023 Aug 24;13(8):e1350. doi: 10.1002/ctm2.1350 (PMC10449817; doi:10.1002/ctm2.1350)
Supplement: Supplementary file 1 — Supporting Information [file CTM2-13-e1350-s001.docx]

**Supporting Information**

**Different solubilizing ability of cyclodextrin derivatives for cholesterol in Niemann–Pick disease type C treatment**

Yusei Yamada^1,2,^*, Madoka Fukaura-Nishizawa^1^, Asami Nishiyama^1^, Akira Ishii^1^, Tatsuya Kawata^1^, Aina Shirakawa^1^, Mayuko Tanaka^1^, Yuki Kondo^1^, Toru Takeo^3^, Naomi Nakagata^4^, Toru Miwa^5^, Hiroki Takeda^6^, Yorihisa Orita^6^, Keiichi Motoyama^7^, Taishi Higashi^8^, Hidetoshi Arima^9^, Takahiro Seki^10^, Yuki Kurauchi^11^, Hiroshi Katsuki^11^, Katsumi Higaki^12^, Kentaro Minami^2^, Naoki Yoshikawa^2^, Ryuji Ikeda^2^, Muneaki Matsuo^13^, Tetsumi Irie^14^, Yoichi Ishitsuka^1,^*

1. Department of Clinical Chemistry and Informatics, Graduate School of Pharmaceutical Sciences, Kumamoto University, 5-1 Oe-honmachi, Chuo-ku, Kumamoto, 862-0973 Japan.
2. Department of Pharmacy, University of Miyazaki Hospital, Miyazaki 889-1692, Japan.
3. Division of Reproductive Engineering, Center for Animal Resources and Development (CARD), Kumamoto University, 2-2-1 Honjo, Chuo-ku, Kumamoto, 860-0811 Japan.
4. Division of Reproductive Biotechnology and Innovation, Center for Animal Resources and Development (CARD), Kumamoto University, 2-2-1 Honjo, Chuo-ku, Kumamoto, 860-0811, Japan.
5. Department of Otolaryngology, Graduate School of Medicine, Osaka Metropolitan University, 1-4-3 Asahi-machi, Abeno-ku, Osaka, 545-8585 Japan.
6. Department of Otolaryngology-Head and Neck Surgery, Graduate School of Medicine, Kumamoto University, 1-1-1 Honjo, Chuo-ku, Kumamoto, 860-0811 Japan.
7. Department of Physical Pharmaceutics, Graduate School of Pharmaceutical Sciences, Kumamoto University, 5-1 Oe-honmachi, Chuo-ku, Kumamoto, 862-0973 Japan.
8. Priority Organization for Innovation and Excellence, Kumamoto University, 5-1 Oe-honmachi, Chuo-ku, Kumamoto 862-0973, Japan.
9. Laboratory of Evidence-Based Pharmacotherapy, Daiichi University of Pharmacy, 22-1 Tamagawa-machi, Minami-ku, Fukuoka, 815-8511 Japan.
10. Department of Pharmacology, Faculty of Pharmaceutical Sciences, Himeji Dokkyo University, 7-2-1 Kami-ohno, Himeji, Hyogo, 670-8524, Japan.
11. Department of Chemico-Pharmacological Sciences, Graduate School of Pharmaceutical Sciences, Kumamoto University, 5-1 Oe-honmachi, Chuo-ku, Kumamoto, 862-0973 Japan.
12. Research Initiative Center, Organization for Research Initiative and Promotion, Tottori University, 86 Nishi-cho, Yonago, 683-8503 Japan.
13. Department of Pediatrics, Faculty of Medicine, Saga University, 5-1-1 Nabeshima, Saga, 849-8501 Japan.
14. Department of Pharmaceutical Packaging Technology, Faculty of Life Sciences, Kumamoto University, 5-1 Oe-honmachi, Chuo-ku, Kumamoto 862-0973, Japan.

*To whom correspondence should be addressed:

Department of Pharmacy, University of Miyazaki Hospital, Miyazaki 889-1692, Japan. Fax: +81- 985-84-3361; Tel: +81-985-85-1512

Yamada Y: [yusei_yamada@med.miyazaki-u.ac.jp](mailto:yusei_yamada@med.miyazaki-u.ac.jp)

Department of Clinical Chemistry and Informatics, Graduate School of Pharmaceutical Sciences, Kumamoto University, 5-1 Oe-honmachi, Chuo-ku, Kumamoto 862-0973, Japan. Fax/Tel: +81-96-371-4559

Ishitsuka Y: [y-zuka@gpo.kumamoto-u.ac.jp](mailto:y-zuka@gpo.kumamoto-u.ac.jp)

**Supplementary Figures**


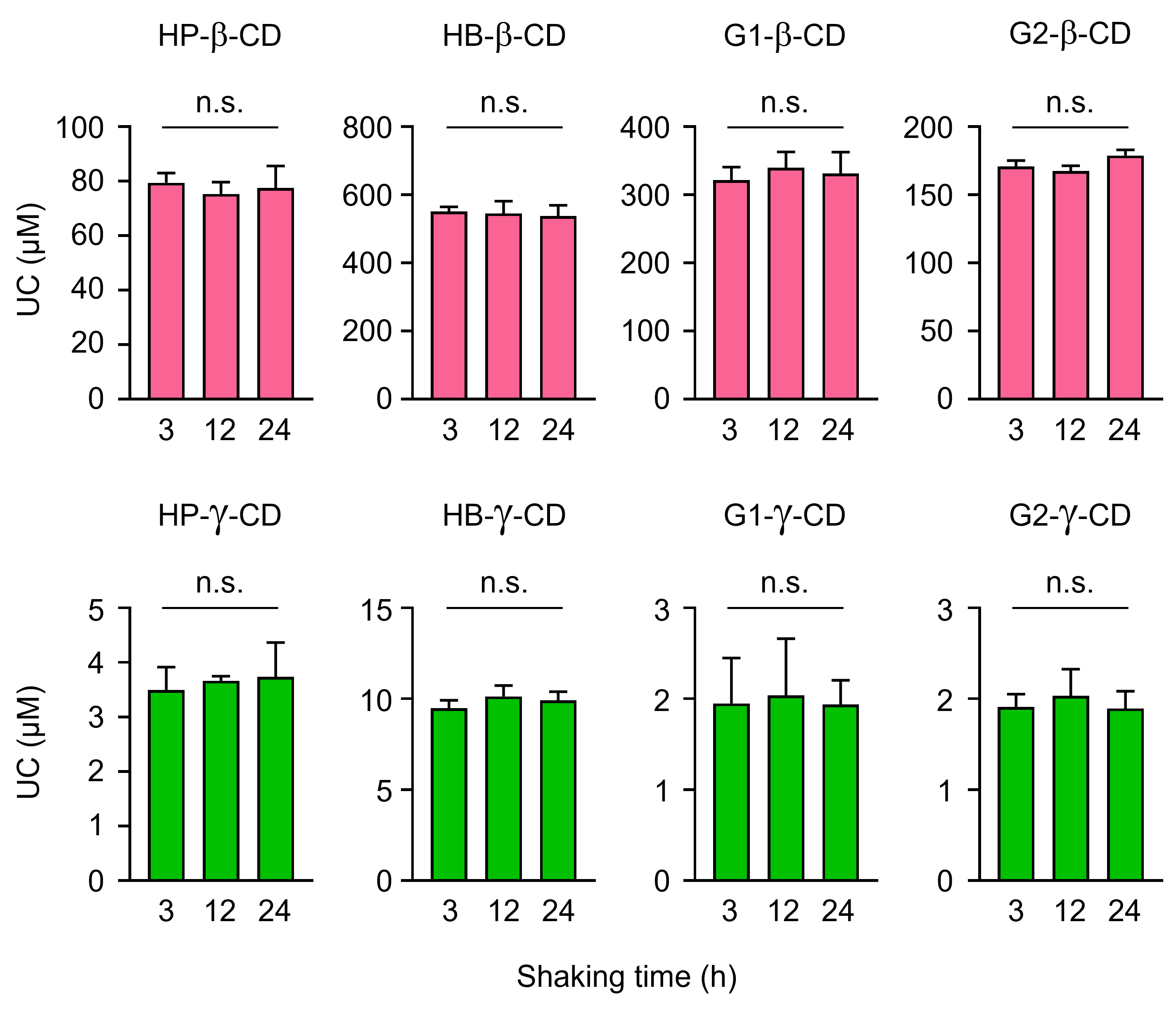


**Supplementary Figure 1. Confirmation of equilibrium in measuring the UC solubility with CD derivatives.**

UC solubilization of CD derivatives in aqueous solution at each shaking time. An excess of UC (10 mg) was added to each concentration of CD derivative in distilled water and shaken at 37 °C for 3, 12, and 24 h at 180 rpm. Data represent the mean ± SEM, n = 3–4. n.s., not significant.


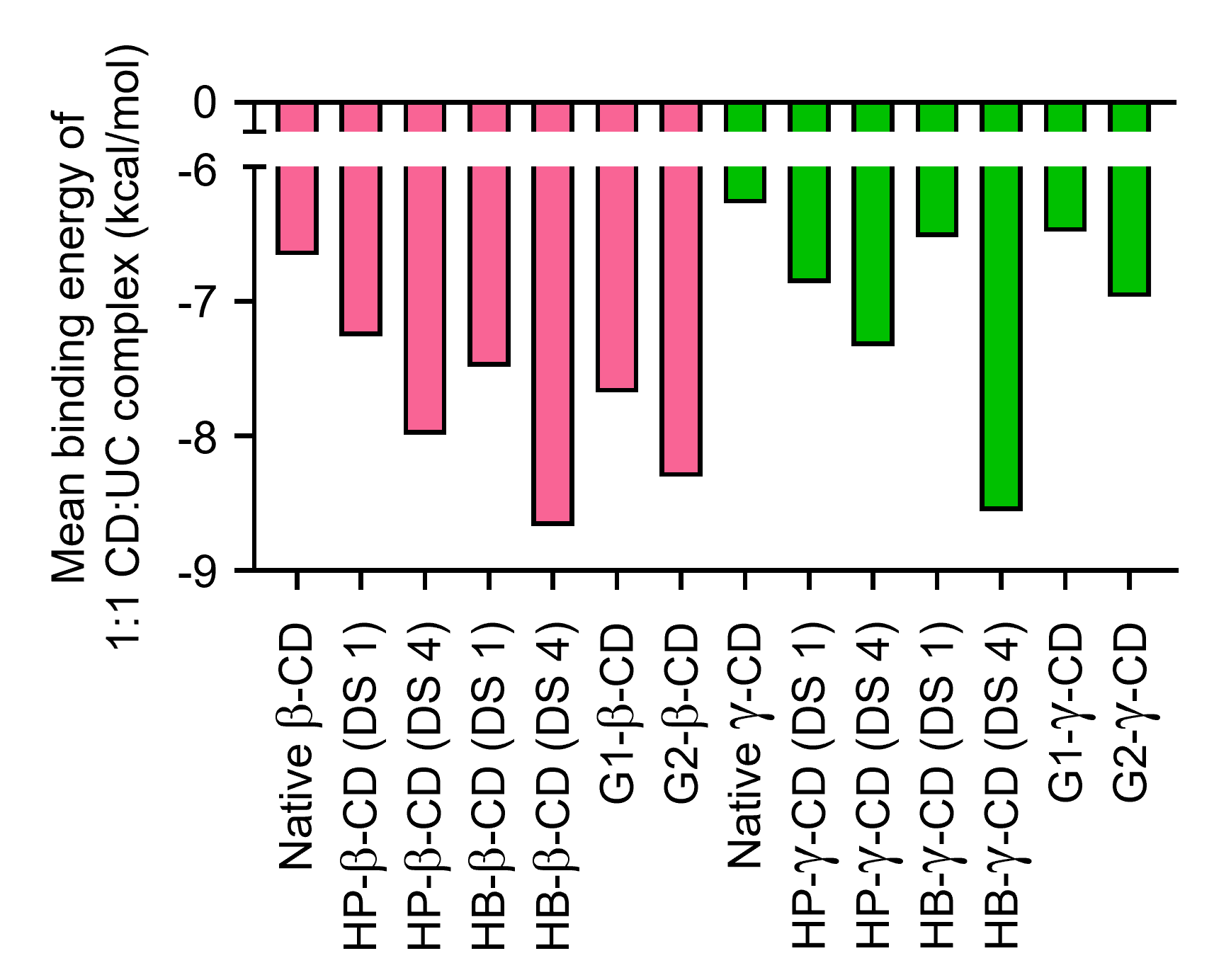
**Supplementary Figure 2. Differences in mean binding energies of 1:1 inclusion complexes of native CDs and their derivatives with UC, associated with the type of substituent and its DS.**

Mean binding energy of 1:1 complex of UC with native CDs and their derivatives, consisting of the single (DS 1)- or multi (DS 4)-hydroxyalkylated CDs and mono-branched CDs calculated from 300 runs with AutoDock.


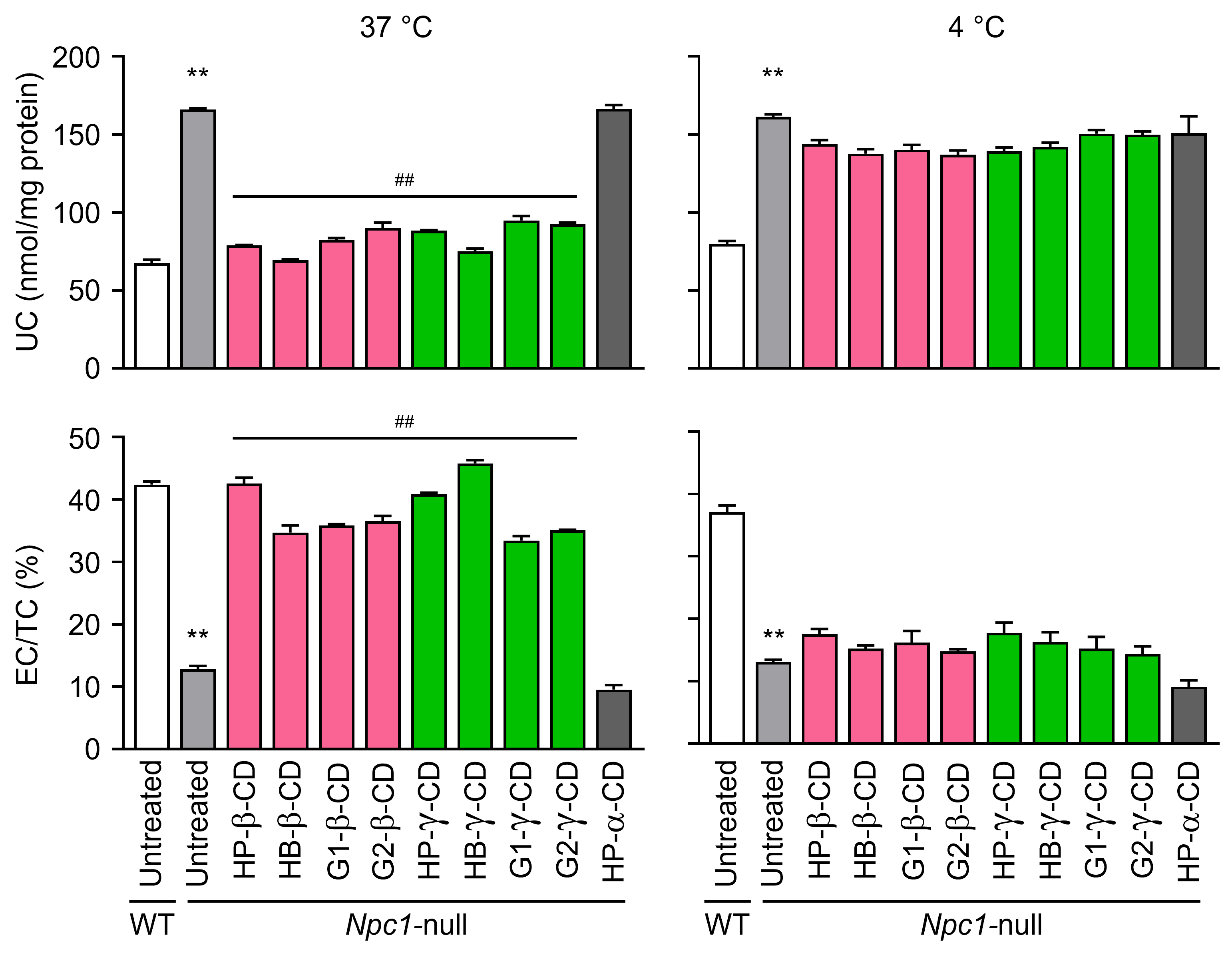


**Supplementary Figure 3.** **Impact of incubation temperature on restorative effect of CD derivatives on intracellular cholesterol balance in NPC model cells.**

Intracellular UC levels (upper panels) and EC/TC ratio (lower panels) in WT and *Npc1*-null cells. Cells were exposed to CD derivatives at 1 mM for 6 h at a physiological temperature (37 °C, left panels) and a low temperature (4 °C, right panels). Cell groups handled at low temperature were preincubated at 4 °C for 2 h and then exposed to cooled CD solution. Data represent the mean ± SEM, n = 3. ^**^*P* < 0.01 vs. untreated WT cells; ^##^*P* < 0.01 vs. untreated *Npc1*-null cells.
